# *catena*-Poly[[[(1,10-phenanthroline-κ^2^ *N*,*N*′)praseodymium(III)]-di-μ-4-hydroxy­benzoato-κ^4^ *O* ^1^:*O* ^1′^-μ-nitrato-κ^3^ *O*,*O*′:*O*] bis­(1,10-phenanthroline)]

**DOI:** 10.1107/S1600536812029911

**Published:** 2012-08-04

**Authors:** Panfeng Wang, Dingding Xu, Xinqing Wang

**Affiliations:** a College of Materials Science and Engineering, China Jiliang University, Hangzhou 310018, People’s Republic of China

## Abstract

The title complex, [Pr(C_7_H_5_O_3_)_2_(NO_3_)(C_12_H_8_N_2_)]·2C_12_H_8_N_2_, has a polymeric chain structure, with two uncoordinated 1,10-phenanthroline mol­ecules in the lattice. The Pr^III^ centre has a monocapped square-anti­prismatic coordination geometry, comprised of two N atoms from one chelating 1,10-phenanthroline ligand, four carboxyl­ate O atoms from four 4-hy­droxy­benzoate anions and three O atoms from two nitrate anions. The 4-hy­droxy­benzoate and nitrate anions function as μ_2_-bridging ligands and link the Pr^III^ ions into a one-dimensional chain structure along the *c* axis. Inter­molecular O—H⋯N hydrogen bonds are observed between the 4-hy­droxy­benzoate anions and the uncoordinated 1,10-phenanthroline mol­ecules.

## Related literature

For related structures, see: Zhou *et al.* (2008 ▶); Zhu *et al.* (2010 ▶)
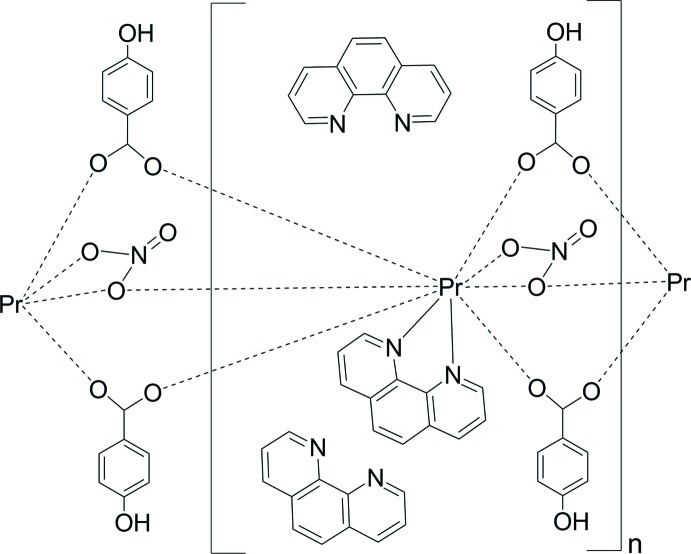


## Experimental

### 

#### Crystal data

[Pr(C_7_H_5_O_3_)_2_(NO_3_)(C_12_H_8_N_2_)]·2C_12_H_8_N_2_
*M* *_r_* = 1017.75
Monoclinic, 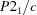

*a* = 21.5625 (2) Å
*b* = 23.4621 (2) Å
*c* = 8.6030 (1) Å
β = 98.899 (1)°
*V* = 4299.88 (7) Å^3^
*Z* = 4
Cu *K*α radiation
μ = 9.27 mm^−1^
*T* = 291 K
0.40 × 0.33 × 0.30 mm

#### Data collection

Oxford Diffraction Gemini S Ultra diffractometer
Absorption correction: multi-scan (*CrysAlis PRO*; Oxford Diffraction, 2006 ▶) *T*_min_ = 0.119, *T*_max_ = 0.167
13264 measured reflections
7308 independent reflections
6794 reflections with *I* > 2σ(*I*)
*R*_int_ = 0.029

#### Refinement

*R*[*F* ^2^ > 2σ(*F* ^2^)] = 0.035
*wR*(*F* ^2^) = 0.092
*S* = 1.06
7308 reflections
606 parameters
H-atom parameters constrained
Δρ_max_ = 0.74 e Å^−3^
Δρ_min_ = −1.31 e Å^−3^

### 

Data collection: *CrysAlis CCD* (Oxford Diffraction, 2006 ▶); cell refinement: *CrysAlis RED* (Oxford Diffraction, 2006 ▶); data reduction: *CrysAlis RED*; program(s) used to solve structure: *SHELXS97* (Sheldrick, 2008 ▶); program(s) used to refine structure: *SHELXL97* (Sheldrick, 2008 ▶); molecular graphics: *ORTEP-3* (Farrugia, 1997 ▶); software used to prepare material for publication: *SHELXL97*.

## Supplementary Material

Crystal structure: contains datablock(s) I, global. DOI: 10.1107/S1600536812029911/fj2550sup1.cif

Structure factors: contains datablock(s) I. DOI: 10.1107/S1600536812029911/fj2550Isup2.hkl

Additional supplementary materials:  crystallographic information; 3D view; checkCIF report

## Figures and Tables

**Table 1:** Hydrogen-bond geometry (Å, °)

*D*—H⋯*A*	*D*—H	H⋯*A*	*D*⋯*A*	*D*—H⋯*A*
O6—H6⋯N4^i^	0.82	2.07	2.851 (4)	158
O3—H3⋯N6^ii^	0.82	2.04	2.782 (5)	150

Symmetry codes: (i) 
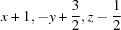
; (ii) 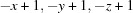.

**Fig. 1.**
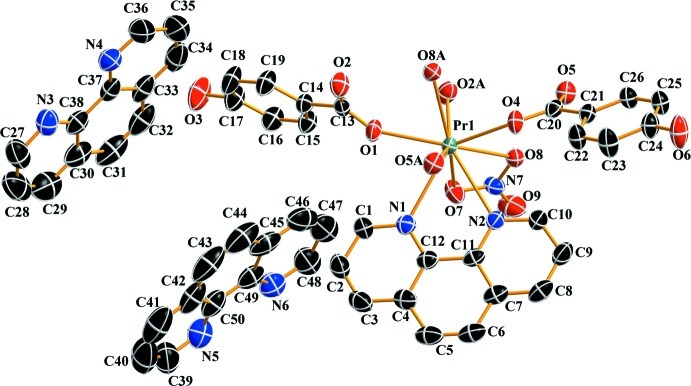
The molecular structure of title compound. Displacement ellipsoids are drawn at the 30% probablility level and H atoms are omitted for clarity.

**Fig. 2.**
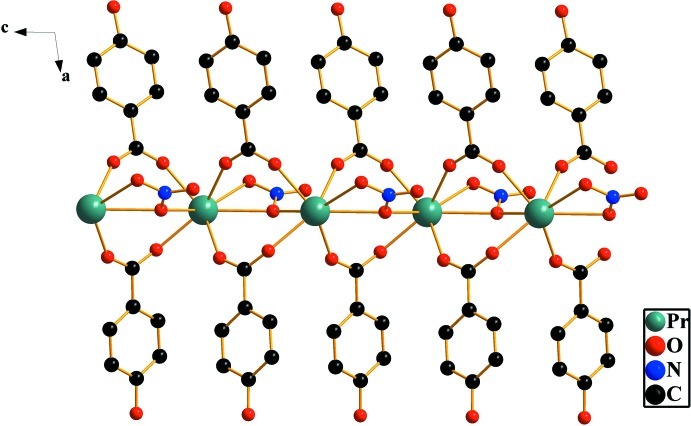
One-dimensional network structure view along b direction.

## supplementary crystallographic information

### Experimental

Each reagent was commercially available and of analytical grade. Pr(NO_3_)_3_^.^6H_2_O (0.217 g, 0.5 mmol), *p*-Hydroxybenzoic acid (0.069 g 0.5 mmol), 1, 10-phenanthroline (0.090 g, 0.5 mmol) and NaHCO_3_ (0.042 g, 0.5 mmol) were dissolved in water-ethanol solution (10 ml, 5:5). The solution was refluxed for 4 h, and filtered after cooling to room temperature. White single crystals were obtained from the filtrate after 7 days.

### Refinement

H atoms were positioned geometrically (C—H = 0.93 Å and O—H = 0.82 Å) and refined as riding, with *U*_iso_ (H) = 1.2Ueq (C) and *U*_iso_(H) = 1.5Ueq (O).

### Crystal data

**Table:**

[Pr(C_7_H_5_O_3_)_2_(NO_3_)(C_12_H_8_N_2_)]·2C_12_H_8_N_2_	*F*(000) = 2056
*M**_r_* = 1017.75	*D*_x_ = 1.572 Mg m^−^^3^
Monoclinic, *P*2_1_/*c*	Cu *K*α radiation, λ = 1.54184 Å
Hall symbol: -P 2ybc	Cell parameters from 9626 reflections
*a* = 21.5625 (2) Å	θ = 1.9–67.4°
*b* = 23.4621 (2) Å	µ = 9.27 mm^−^^1^
*c* = 8.6030 (1) Å	*T* = 291 K
β = 98.899 (1)°	Needle, white
*V* = 4299.88 (7) Å^3^	0.40 × 0.33 × 0.30 mm
*Z* = 4	

### Data collection

**Table:**

Oxford Diffraction Gemini S Ultra diffractometer	7308 independent reflections
Radiation source: Enhance Ultra (Cu) X-ray Source	6794 reflections with *I* > 2σ(*I*)
Mirror monochromator	*R*_int_ = 0.029
Detector resolution: 15.9149 pixels mm^-1^	θ_max_ = 65.1°, θ_min_ = 2.1°
ω scans	*h* = −25→25
Absorption correction: multi-scan (*CrysAlis PRO*; Oxford Diffraction, 2006)	*k* = −27→16
*T*_min_ = 0.119, *T*_max_ = 0.167	*l* = −9→10
13264 measured reflections	

### Refinement

**Table:**

Refinement on *F*^2^	Primary atom site location: structure-invariant direct methods
Least-squares matrix: full	Secondary atom site location: difference Fourier map
*R*[*F*^2^ > 2σ(*F*^2^)] = 0.035	Hydrogen site location: inferred from neighbouring sites
*wR*(*F*^2^) = 0.092	H-atom parameters constrained
*S* = 1.06	*w* = 1/[σ^2^(*F*_o_^2^) + (0.060*P*)^2^] where *P* = (*F*_o_^2^ + 2*F*_c_^2^)/3
7308 reflections	(Δ/σ)_max_ = 0.001
606 parameters	Δρ_max_ = 0.74 e Å^−^^3^
0 restraints	Δρ_min_ = −1.31 e Å^−^^3^

### Special details

**Table:**

Experimental. CrysAlisPro, Oxford Diffraction Ltd., Version 1.171.34.36 Empirical absorption correction using spherical harmonics, implemented in SCALE3 ABSPACK scaling algorithm.
Geometry. All e.s.d.'s (except the e.s.d. in the dihedral angle between two l.s. planes) are estimated using the full covariance matrix. The cell e.s.d.'s are taken into account individually in the estimation of e.s.d.'s in distances, angles and torsion angles; correlations between e.s.d.'s in cell parameters are only used when they are defined by crystal symmetry. An approximate (isotropic) treatment of cell e.s.d.'s is used for estimating e.s.d.'s involving l.s. planes.
Refinement. Refinement of *F*^2^ against ALL reflections. The weighted *R*-factor *wR* and goodness of fit *S* are based on *F*^2^, conventional *R*-factors *R* are based on *F*, with *F* set to zero for negative *F*^2^. The threshold expression of *F*^2^ > σ(*F*^2^) is used only for calculating *R*-factors(gt) *etc*. and is not relevant to the choice of reflections for refinement. *R*-factors based on *F*^2^ are statistically about twice as large as those based on *F*, and *R*- factors based on ALL data will be even larger.

### Fractional atomic coordinates and isotropic or equivalent isotropic displacement parameters (Å^2^)

**Table:**

	*x*	*y*	*z*	*U*_iso_*/*U*_eq_	
Pr1	0.911380 (7)	0.703539 (6)	0.660845 (17)	0.02673 (8)	
O1	0.82724 (10)	0.69075 (10)	0.8105 (3)	0.0402 (5)	
O2	0.82646 (10)	0.74065 (11)	1.0300 (3)	0.0446 (5)	
O3	0.54691 (13)	0.65243 (17)	0.9284 (4)	0.0741 (10)	
H3	0.5307	0.6376	0.8457	0.111*	
O4	0.99741 (10)	0.76381 (10)	0.6186 (3)	0.0388 (5)	
O5	0.98379 (10)	0.81352 (10)	0.3967 (3)	0.0393 (5)	
O6	1.27882 (11)	0.83426 (15)	0.5929 (3)	0.0609 (8)	
H6	1.2891	0.8501	0.5159	0.091*	
O7	0.85863 (12)	0.63832 (10)	0.4358 (3)	0.0473 (6)	
O8	0.91180 (11)	0.70593 (8)	0.3492 (3)	0.0344 (5)	
O9	0.87386 (16)	0.63694 (11)	0.1925 (3)	0.0620 (8)	
N1	0.91051 (12)	0.58977 (11)	0.7443 (3)	0.0372 (6)	
N2	0.99832 (12)	0.63129 (11)	0.5726 (3)	0.0360 (6)	
N3	0.29434 (16)	0.53994 (16)	0.9226 (4)	0.0588 (8)	
N4	0.32464 (19)	0.64182 (15)	0.8063 (4)	0.0628 (9)	
N5	0.50294 (18)	0.33752 (18)	0.4667 (5)	0.0684 (10)	
N6	0.52702 (16)	0.42317 (16)	0.2691 (4)	0.0608 (9)	
N7	0.88073 (13)	0.65978 (11)	0.3208 (3)	0.0382 (6)	
C1	0.86749 (17)	0.56790 (15)	0.8203 (4)	0.0452 (8)	
H1	0.8333	0.5905	0.8339	0.054*	
C2	0.8705 (2)	0.51245 (17)	0.8815 (5)	0.0570 (10)	
H2	0.8385	0.4987	0.9324	0.068*	
C3	0.9202 (2)	0.47938 (16)	0.8658 (4)	0.0560 (10)	
H3A	0.9230	0.4426	0.9064	0.067*	
C4	0.96762 (18)	0.50076 (15)	0.7881 (4)	0.0455 (8)	
C5	1.0222 (2)	0.46801 (16)	0.7694 (4)	0.0542 (9)	
H5	1.0271	0.4316	0.8125	0.065*	
C6	1.06627 (19)	0.48895 (16)	0.6909 (5)	0.0532 (9)	
H6A	1.1014	0.4670	0.6810	0.064*	
C7	1.05998 (16)	0.54452 (15)	0.6224 (4)	0.0450 (8)	
C8	1.10422 (18)	0.56691 (18)	0.5375 (5)	0.0554 (10)	
H8	1.1401	0.5461	0.5269	0.066*	
C9	1.09484 (17)	0.61956 (18)	0.4694 (5)	0.0541 (9)	
H9	1.1240	0.6349	0.4119	0.065*	
C10	1.04075 (16)	0.64978 (15)	0.4880 (4)	0.0440 (8)	
H10	1.0340	0.6849	0.4382	0.053*	
C11	1.00721 (15)	0.57883 (13)	0.6386 (4)	0.0360 (7)	
C12	0.96066 (15)	0.55657 (13)	0.7261 (4)	0.0368 (7)	
C13	0.80099 (15)	0.70958 (12)	0.9199 (4)	0.0322 (7)	
C14	0.73337 (15)	0.69387 (13)	0.9191 (4)	0.0334 (6)	
C15	0.69901 (15)	0.66640 (15)	0.7909 (4)	0.0393 (7)	
H15	0.7182	0.6573	0.7044	0.047*	
C16	0.63630 (16)	0.65238 (16)	0.7902 (4)	0.0470 (8)	
H16	0.6135	0.6347	0.7028	0.056*	
C17	0.60788 (16)	0.66489 (18)	0.9204 (5)	0.0517 (9)	
C18	0.64241 (19)	0.6915 (2)	1.0494 (5)	0.0602 (11)	
H18	0.6239	0.6994	1.1377	0.072*	
C19	0.70412 (18)	0.70630 (15)	1.0472 (5)	0.0475 (9)	
H19	0.7265	0.7250	1.1335	0.057*	
C20	1.01715 (15)	0.79388 (12)	0.5157 (4)	0.0317 (7)	
C21	1.08667 (14)	0.80619 (13)	0.5374 (4)	0.0312 (6)	
C22	1.12756 (16)	0.77961 (17)	0.6560 (4)	0.0457 (8)	
H22	1.1119	0.7551	0.7255	0.055*	
C23	1.19154 (18)	0.78935 (18)	0.6716 (5)	0.0543 (10)	
H23	1.2185	0.7713	0.7513	0.065*	
C24	1.21567 (15)	0.82586 (16)	0.5690 (4)	0.0432 (8)	
C25	1.17529 (15)	0.85264 (15)	0.4508 (4)	0.0409 (7)	
H25	1.1908	0.8776	0.3822	0.049*	
C26	1.11146 (15)	0.84181 (14)	0.4357 (4)	0.0371 (7)	
H26	1.0846	0.8591	0.3545	0.045*	
C27	0.2817 (2)	0.4893 (2)	0.9770 (7)	0.0762 (14)	
H27	0.2878	0.4838	1.0853	0.091*	
C28	0.2599 (3)	0.4442 (3)	0.8801 (10)	0.097 (2)	
H28	0.2521	0.4092	0.9237	0.116*	
C29	0.2501 (2)	0.4509 (3)	0.7224 (9)	0.0909 (18)	
H29	0.2343	0.4209	0.6573	0.109*	
C30	0.26354 (17)	0.5030 (2)	0.6578 (6)	0.0656 (12)	
C31	0.2559 (2)	0.5159 (3)	0.4895 (6)	0.0795 (17)	
H31	0.2396	0.4880	0.4178	0.095*	
C32	0.2714 (2)	0.5660 (3)	0.4363 (6)	0.0729 (14)	
H32	0.2657	0.5724	0.3283	0.087*	
C33	0.29633 (18)	0.6099 (2)	0.5385 (5)	0.0578 (11)	
C34	0.3163 (2)	0.6624 (2)	0.4868 (6)	0.0741 (14)	
H34	0.3126	0.6700	0.3796	0.089*	
C35	0.3411 (4)	0.7024 (2)	0.5929 (8)	0.093 (2)	
H35	0.3564	0.7368	0.5604	0.112*	
C36	0.3430 (3)	0.6902 (2)	0.7513 (7)	0.0863 (17)	
H36	0.3583	0.7182	0.8237	0.104*	
C37	0.30263 (17)	0.60086 (18)	0.7031 (4)	0.0505 (9)	
C38	0.28600 (16)	0.54654 (18)	0.7640 (5)	0.0508 (9)	
C39	0.4948 (3)	0.2955 (2)	0.5625 (8)	0.0887 (18)	
H39	0.4729	0.2635	0.5204	0.106*	
C40	0.5173 (4)	0.2960 (3)	0.7245 (9)	0.108 (3)	
H40	0.5115	0.2648	0.7873	0.130*	
C41	0.5475 (3)	0.3430 (4)	0.7867 (7)	0.103 (2)	
H41	0.5621	0.3446	0.8942	0.124*	
C42	0.5571 (2)	0.3895 (3)	0.6909 (6)	0.0754 (15)	
C43	0.5878 (2)	0.4410 (4)	0.7490 (7)	0.095 (2)	
H43	0.6026	0.4443	0.8560	0.113*	
C44	0.5958 (2)	0.4845 (3)	0.6533 (8)	0.092 (2)	
H44	0.6146	0.5179	0.6955	0.110*	
C45	0.57586 (18)	0.4804 (2)	0.4878 (7)	0.0728 (14)	
C46	0.5861 (2)	0.5236 (2)	0.3827 (9)	0.0855 (17)	
H46	0.6047	0.5577	0.4203	0.103*	
C47	0.5686 (2)	0.5157 (3)	0.2249 (10)	0.0897 (18)	
H47	0.5763	0.5434	0.1529	0.108*	
C48	0.5387 (2)	0.4646 (2)	0.1750 (7)	0.0743 (13)	
H48	0.5262	0.4595	0.0676	0.089*	
C49	0.54567 (17)	0.42982 (19)	0.4263 (5)	0.0579 (10)	
C50	0.53442 (17)	0.3839 (2)	0.5292 (5)	0.0589 (10)	

### Atomic displacement parameters (Å^2^)

**Table:**

	*U*^11^	*U*^22^	*U*^33^	*U*^12^	*U*^13^	*U*^23^
Pr1	0.02430 (11)	0.03072 (12)	0.02603 (11)	0.00020 (5)	0.00666 (7)	−0.00008 (5)
O1	0.0344 (11)	0.0491 (12)	0.0408 (13)	−0.0055 (10)	0.0173 (10)	−0.0017 (10)
O2	0.0381 (12)	0.0556 (14)	0.0408 (13)	−0.0136 (11)	0.0080 (10)	−0.0071 (11)
O3	0.0370 (14)	0.111 (3)	0.078 (2)	−0.0256 (15)	0.0191 (14)	−0.0280 (19)
O4	0.0333 (10)	0.0483 (13)	0.0367 (12)	−0.0096 (9)	0.0113 (9)	0.0016 (10)
O5	0.0331 (11)	0.0512 (13)	0.0323 (11)	−0.0029 (10)	0.0008 (9)	0.0005 (10)
O6	0.0308 (12)	0.102 (2)	0.0493 (15)	−0.0079 (13)	0.0055 (11)	0.0225 (15)
O7	0.0578 (14)	0.0437 (13)	0.0404 (13)	−0.0166 (11)	0.0074 (11)	0.0008 (10)
O8	0.0381 (12)	0.0333 (12)	0.0330 (12)	−0.0010 (8)	0.0090 (10)	0.0008 (8)
O9	0.100 (2)	0.0472 (14)	0.0361 (13)	0.0003 (15)	0.0019 (14)	−0.0124 (11)
N1	0.0403 (14)	0.0380 (14)	0.0334 (13)	0.0007 (11)	0.0057 (11)	−0.0008 (11)
N2	0.0361 (13)	0.0383 (14)	0.0349 (13)	0.0030 (11)	0.0101 (11)	−0.0013 (11)
N3	0.0498 (18)	0.071 (2)	0.057 (2)	0.0027 (16)	0.0108 (15)	0.0065 (17)
N4	0.085 (3)	0.055 (2)	0.0530 (19)	0.0041 (18)	0.0268 (19)	−0.0061 (16)
N5	0.059 (2)	0.086 (3)	0.060 (2)	−0.004 (2)	0.0087 (18)	−0.003 (2)
N6	0.0463 (17)	0.068 (2)	0.066 (2)	0.0057 (16)	0.0029 (16)	−0.0012 (18)
N7	0.0494 (15)	0.0292 (13)	0.0352 (15)	−0.0003 (12)	0.0043 (12)	−0.0014 (11)
C1	0.0495 (19)	0.0416 (18)	0.0469 (19)	−0.0023 (15)	0.0156 (16)	0.0044 (15)
C2	0.071 (3)	0.052 (2)	0.052 (2)	−0.0089 (19)	0.023 (2)	0.0068 (17)
C3	0.083 (3)	0.0395 (18)	0.046 (2)	0.0005 (19)	0.012 (2)	0.0078 (16)
C4	0.062 (2)	0.0403 (17)	0.0318 (16)	0.0057 (16)	0.0014 (15)	0.0013 (14)
C5	0.077 (3)	0.0399 (18)	0.0433 (19)	0.0168 (19)	0.0006 (19)	0.0007 (15)
C6	0.059 (2)	0.047 (2)	0.051 (2)	0.0202 (17)	0.0000 (18)	−0.0073 (17)
C7	0.0457 (18)	0.0481 (19)	0.0394 (17)	0.0121 (15)	0.0011 (15)	−0.0086 (15)
C8	0.0423 (18)	0.063 (2)	0.061 (2)	0.0181 (17)	0.0112 (17)	−0.0090 (19)
C9	0.0437 (19)	0.062 (2)	0.062 (2)	0.0063 (17)	0.0236 (18)	−0.0072 (19)
C10	0.0434 (18)	0.0438 (18)	0.0481 (19)	0.0003 (14)	0.0171 (15)	−0.0040 (15)
C11	0.0368 (15)	0.0385 (17)	0.0315 (15)	0.0068 (13)	0.0013 (12)	−0.0072 (13)
C12	0.0434 (16)	0.0364 (16)	0.0294 (15)	0.0030 (13)	0.0026 (13)	−0.0046 (12)
C13	0.0292 (15)	0.0345 (15)	0.0343 (17)	−0.0015 (11)	0.0088 (13)	0.0078 (12)
C14	0.0297 (15)	0.0376 (15)	0.0339 (16)	−0.0031 (12)	0.0080 (13)	−0.0012 (12)
C15	0.0346 (15)	0.0486 (18)	0.0353 (16)	−0.0042 (14)	0.0071 (13)	−0.0068 (14)
C16	0.0372 (17)	0.057 (2)	0.0459 (19)	−0.0091 (15)	0.0035 (15)	−0.0128 (16)
C17	0.0328 (16)	0.066 (2)	0.057 (2)	−0.0124 (16)	0.0119 (16)	−0.0113 (18)
C18	0.043 (2)	0.087 (3)	0.056 (2)	−0.013 (2)	0.0251 (19)	−0.024 (2)
C19	0.0377 (19)	0.065 (2)	0.041 (2)	−0.0108 (15)	0.0098 (16)	−0.0185 (15)
C20	0.0310 (16)	0.0336 (16)	0.0319 (16)	−0.0030 (11)	0.0090 (13)	−0.0083 (12)
C21	0.0299 (15)	0.0368 (15)	0.0277 (15)	−0.0026 (12)	0.0064 (12)	−0.0029 (12)
C22	0.0383 (17)	0.058 (2)	0.0409 (18)	−0.0041 (16)	0.0072 (15)	0.0152 (17)
C23	0.0362 (19)	0.079 (3)	0.047 (2)	−0.0011 (17)	0.0010 (16)	0.0241 (18)
C24	0.0317 (15)	0.059 (2)	0.0392 (17)	−0.0079 (15)	0.0059 (13)	0.0031 (15)
C25	0.0370 (16)	0.0492 (19)	0.0370 (17)	−0.0066 (14)	0.0071 (14)	0.0091 (14)
C26	0.0331 (15)	0.0425 (17)	0.0355 (16)	−0.0029 (13)	0.0047 (13)	0.0047 (13)
C27	0.061 (3)	0.077 (3)	0.092 (4)	−0.006 (2)	0.016 (3)	0.024 (3)
C28	0.065 (3)	0.074 (4)	0.154 (6)	−0.010 (3)	0.026 (4)	0.021 (4)
C29	0.055 (3)	0.084 (4)	0.134 (5)	−0.019 (3)	0.014 (3)	−0.034 (4)
C30	0.0308 (17)	0.077 (3)	0.089 (3)	−0.0004 (19)	0.0083 (19)	−0.024 (2)
C31	0.040 (2)	0.117 (5)	0.078 (3)	0.006 (2)	−0.002 (2)	−0.049 (3)
C32	0.045 (2)	0.118 (4)	0.055 (3)	0.015 (3)	0.0059 (19)	−0.018 (3)
C33	0.0412 (19)	0.088 (3)	0.046 (2)	0.021 (2)	0.0110 (16)	−0.002 (2)
C34	0.072 (3)	0.099 (4)	0.056 (3)	0.028 (3)	0.026 (2)	0.023 (3)
C35	0.133 (6)	0.070 (4)	0.086 (4)	0.017 (3)	0.047 (4)	0.018 (3)
C36	0.130 (5)	0.056 (3)	0.082 (4)	0.001 (3)	0.047 (4)	−0.002 (3)
C37	0.0444 (18)	0.067 (2)	0.0424 (19)	0.0175 (17)	0.0140 (15)	0.0004 (17)
C38	0.0319 (16)	0.066 (2)	0.056 (2)	0.0061 (16)	0.0088 (15)	−0.0082 (18)
C39	0.088 (4)	0.094 (4)	0.089 (4)	−0.003 (3)	0.029 (4)	0.014 (3)
C40	0.112 (6)	0.137 (7)	0.083 (5)	0.021 (4)	0.039 (4)	0.035 (4)
C41	0.084 (4)	0.169 (7)	0.060 (3)	0.034 (4)	0.019 (3)	−0.001 (4)
C42	0.047 (2)	0.124 (5)	0.056 (3)	0.020 (3)	0.011 (2)	−0.017 (3)
C43	0.053 (3)	0.157 (6)	0.073 (4)	0.010 (3)	0.006 (2)	−0.056 (4)
C44	0.050 (3)	0.122 (5)	0.105 (4)	0.000 (3)	0.011 (3)	−0.061 (4)
C45	0.0330 (19)	0.081 (3)	0.103 (4)	0.012 (2)	0.008 (2)	−0.037 (3)
C46	0.048 (3)	0.061 (3)	0.150 (6)	0.005 (2)	0.024 (3)	−0.013 (4)
C47	0.050 (3)	0.082 (4)	0.140 (6)	0.013 (2)	0.023 (3)	0.022 (4)
C48	0.054 (2)	0.077 (3)	0.092 (4)	0.007 (2)	0.009 (2)	0.015 (3)
C49	0.0313 (16)	0.071 (3)	0.070 (3)	0.0110 (17)	0.0049 (17)	−0.016 (2)
C50	0.0372 (18)	0.087 (3)	0.053 (2)	0.0113 (19)	0.0060 (16)	−0.011 (2)

### Geometric parameters (Å, º)

**Table:**

Pr1—O2^i^	2.387 (2)	C15—H15	0.9300
Pr1—O5^ii^	2.395 (2)	C16—C17	1.389 (5)
Pr1—O1	2.400 (2)	C16—H16	0.9300
Pr1—O4	2.404 (2)	C17—C18	1.385 (6)
Pr1—O7	2.589 (2)	C18—C19	1.378 (5)
Pr1—O8^ii^	2.671 (2)	C18—H18	0.9300
Pr1—O8	2.683 (2)	C19—H19	0.9300
Pr1—N2	2.721 (3)	C20—C21	1.509 (4)
Pr1—N1	2.765 (3)	C21—C26	1.376 (5)
O1—C13	1.251 (4)	C21—C22	1.389 (5)
O2—C13	1.253 (4)	C22—C23	1.384 (5)
O2—Pr1^ii^	2.387 (2)	C22—H22	0.9300
O3—C17	1.359 (4)	C23—C24	1.388 (5)
O3—H3	0.8200	C23—H23	0.9300
O4—C20	1.257 (4)	C24—C25	1.383 (5)
O5—C20	1.245 (4)	C25—C26	1.386 (4)
O5—Pr1^i^	2.395 (2)	C25—H25	0.9300
O6—C24	1.360 (4)	C26—H26	0.9300
O6—H6	0.8200	C27—C28	1.385 (9)
O7—N7	1.267 (4)	C27—H27	0.9300
O8—N7	1.277 (3)	C28—C29	1.349 (9)
O8—Pr1^i^	2.671 (2)	C28—H28	0.9300
O9—N7	1.215 (4)	C29—C30	1.392 (8)
N1—C1	1.318 (4)	C29—H29	0.9300
N1—C12	1.361 (4)	C30—C38	1.407 (6)
N2—C10	1.327 (4)	C30—C31	1.463 (7)
N2—C11	1.357 (4)	C31—C32	1.325 (8)
N3—C27	1.320 (6)	C31—H31	0.9300
N3—C38	1.357 (5)	C32—C33	1.407 (7)
N4—C36	1.314 (6)	C32—H32	0.9300
N4—C37	1.344 (5)	C33—C34	1.399 (7)
N5—C39	1.314 (7)	C33—C37	1.418 (5)
N5—C50	1.350 (6)	C34—C35	1.360 (9)
N6—C48	1.314 (6)	C34—H34	0.9300
N6—C49	1.359 (6)	C35—C36	1.387 (8)
C1—C2	1.401 (5)	C35—H35	0.9300
C1—H1	0.9300	C36—H36	0.9300
C2—C3	1.347 (6)	C37—C38	1.444 (6)
C2—H2	0.9300	C39—C40	1.403 (10)
C3—C4	1.399 (6)	C39—H39	0.9300
C3—H3A	0.9300	C40—C41	1.350 (10)
C4—C12	1.413 (5)	C40—H40	0.9300
C4—C5	1.436 (5)	C41—C42	1.402 (10)
C5—C6	1.341 (6)	C41—H41	0.9300
C5—H5	0.9300	C42—C50	1.407 (6)
C6—C7	1.429 (5)	C42—C43	1.429 (9)
C6—H6A	0.9300	C43—C44	1.340 (10)
C7—C8	1.391 (6)	C43—H43	0.9300
C7—C11	1.418 (4)	C44—C45	1.425 (8)
C8—C9	1.368 (6)	C44—H44	0.9300
C8—H8	0.9300	C45—C46	1.399 (8)
C9—C10	1.395 (5)	C45—C49	1.416 (6)
C9—H9	0.9300	C46—C47	1.364 (9)
C10—H10	0.9300	C46—H46	0.9300
C11—C12	1.442 (5)	C47—C48	1.397 (8)
C13—C14	1.503 (4)	C47—H47	0.9300
C14—C19	1.383 (5)	C48—H48	0.9300
C14—C15	1.388 (5)	C49—C50	1.438 (7)
C15—C16	1.391 (5)		
			
O2^i^—Pr1—O5^ii^	147.50 (8)	C17—C16—H16	120.1
O2^i^—Pr1—O1	74.53 (8)	C15—C16—H16	120.1
O5^ii^—Pr1—O1	88.43 (8)	O3—C17—C18	117.4 (3)
O2^i^—Pr1—O4	99.18 (8)	O3—C17—C16	123.1 (3)
O5^ii^—Pr1—O4	79.01 (8)	C18—C17—C16	119.4 (3)
O1—Pr1—O4	145.03 (8)	C19—C18—C17	120.2 (3)
O2^i^—Pr1—O7	75.83 (9)	C19—C18—H18	119.9
O5^ii^—Pr1—O7	133.49 (8)	C17—C18—H18	119.9
O1—Pr1—O7	92.65 (8)	C18—C19—C14	121.2 (3)
O4—Pr1—O7	119.63 (7)	C18—C19—H19	119.4
O2^i^—Pr1—O8^ii^	77.31 (8)	C14—C19—H19	119.4
O5^ii^—Pr1—O8^ii^	71.23 (8)	O5—C20—O4	124.8 (3)
O1—Pr1—O8^ii^	72.90 (7)	O5—C20—C21	117.9 (3)
O4—Pr1—O8^ii^	72.17 (7)	O4—C20—C21	117.3 (3)
O7—Pr1—O8^ii^	152.09 (8)	C26—C21—C22	118.3 (3)
O2^i^—Pr1—O8	68.78 (7)	C26—C21—C20	120.9 (3)
O5^ii^—Pr1—O8	138.38 (7)	C22—C21—C20	120.7 (3)
O1—Pr1—O8	131.16 (8)	C23—C22—C21	120.4 (3)
O4—Pr1—O8	73.42 (7)	C23—C22—H22	119.8
O7—Pr1—O8	48.24 (7)	C21—C22—H22	119.8
O8^ii^—Pr1—O8	126.12 (4)	C22—C23—C24	120.4 (3)
O2^i^—Pr1—N2	136.17 (8)	C22—C23—H23	119.8
O5^ii^—Pr1—N2	75.24 (8)	C24—C23—H23	119.8
O1—Pr1—N2	133.02 (8)	O6—C24—C25	123.2 (3)
O4—Pr1—N2	75.13 (8)	O6—C24—C23	117.3 (3)
O7—Pr1—N2	70.43 (8)	C25—C24—C23	119.5 (3)
O8^ii^—Pr1—N2	136.62 (8)	C24—C25—C26	119.3 (3)
O8—Pr1—N2	67.99 (7)	C24—C25—H25	120.4
O2^i^—Pr1—N1	128.07 (8)	C26—C25—H25	120.4
O5^ii^—Pr1—N1	69.41 (8)	C21—C26—C25	122.0 (3)
O1—Pr1—N1	72.78 (8)	C21—C26—H26	119.0
O4—Pr1—N1	130.05 (8)	C25—C26—H26	119.0
O7—Pr1—N1	66.66 (8)	N3—C27—C28	123.0 (5)
O8^ii^—Pr1—N1	127.56 (7)	N3—C27—H27	118.5
O8—Pr1—N1	106.31 (7)	C28—C27—H27	118.5
N2—Pr1—N1	60.24 (8)	C29—C28—C27	120.1 (6)
C13—O1—Pr1	145.7 (2)	C29—C28—H28	119.9
C13—O2—Pr1^ii^	152.9 (2)	C27—C28—H28	119.9
C17—O3—H3	109.5	C28—C29—C30	119.6 (5)
C20—O4—Pr1	142.2 (2)	C28—C29—H29	120.2
C20—O5—Pr1^i^	148.5 (2)	C30—C29—H29	120.2
C24—O6—H6	109.5	C29—C30—C38	116.8 (5)
N7—O7—Pr1	100.04 (17)	C29—C30—C31	125.3 (5)
N7—O8—Pr1^i^	127.27 (19)	C38—C30—C31	117.9 (5)
N7—O8—Pr1	95.22 (16)	C32—C31—C30	122.0 (4)
Pr1^i^—O8—Pr1	128.52 (8)	C32—C31—H31	119.0
C1—N1—C12	117.8 (3)	C30—C31—H31	119.0
C1—N1—Pr1	122.5 (2)	C31—C32—C33	121.8 (5)
C12—N1—Pr1	119.0 (2)	C31—C32—H32	119.1
C10—N2—C11	117.5 (3)	C33—C32—H32	119.1
C10—N2—Pr1	120.8 (2)	C34—C33—C32	123.5 (4)
C11—N2—Pr1	120.47 (19)	C34—C33—C37	117.6 (4)
C27—N3—C38	117.2 (4)	C32—C33—C37	118.9 (5)
C36—N4—C37	118.4 (4)	C35—C34—C33	120.1 (4)
C39—N5—C50	117.6 (5)	C35—C34—H34	119.9
C48—N6—C49	118.2 (4)	C33—C34—H34	119.9
O9—N7—O7	121.7 (3)	C34—C35—C36	117.8 (5)
O9—N7—O8	122.3 (3)	C34—C35—H35	121.1
O7—N7—O8	115.9 (3)	C36—C35—H35	121.1
N1—C1—C2	123.5 (3)	N4—C36—C35	124.5 (6)
N1—C1—H1	118.2	N4—C36—H36	117.7
C2—C1—H1	118.2	C35—C36—H36	117.7
C3—C2—C1	119.2 (4)	N4—C37—C33	121.4 (4)
C3—C2—H2	120.4	N4—C37—C38	118.3 (3)
C1—C2—H2	120.4	C33—C37—C38	120.3 (4)
C2—C3—C4	119.6 (3)	N3—C38—C30	123.3 (4)
C2—C3—H3A	120.2	N3—C38—C37	117.6 (4)
C4—C3—H3A	120.2	C30—C38—C37	119.1 (4)
C3—C4—C12	118.0 (3)	N5—C39—C40	123.9 (6)
C3—C4—C5	122.3 (3)	N5—C39—H39	118.0
C12—C4—C5	119.7 (3)	C40—C39—H39	118.0
C6—C5—C4	121.1 (3)	C41—C40—C39	118.2 (6)
C6—C5—H5	119.4	C41—C40—H40	120.9
C4—C5—H5	119.4	C39—C40—H40	120.9
C5—C6—C7	120.9 (3)	C40—C41—C42	120.6 (6)
C5—C6—H6A	119.6	C40—C41—H41	119.7
C7—C6—H6A	119.6	C42—C41—H41	119.7
C8—C7—C11	117.6 (3)	C41—C42—C50	116.6 (6)
C8—C7—C6	122.2 (3)	C41—C42—C43	123.7 (6)
C11—C7—C6	120.2 (3)	C50—C42—C43	119.7 (6)
C9—C8—C7	119.8 (3)	C44—C43—C42	121.7 (5)
C9—C8—H8	120.1	C44—C43—H43	119.1
C7—C8—H8	120.1	C42—C43—H43	119.1
C8—C9—C10	118.7 (4)	C43—C44—C45	121.0 (6)
C8—C9—H9	120.6	C43—C44—H44	119.5
C10—C9—H9	120.6	C45—C44—H44	119.5
N2—C10—C9	123.8 (3)	C46—C45—C49	118.4 (5)
N2—C10—H10	118.1	C46—C45—C44	122.9 (6)
C9—C10—H10	118.1	C49—C45—C44	118.7 (6)
N2—C11—C7	122.5 (3)	C47—C46—C45	119.9 (5)
N2—C11—C12	118.8 (3)	C47—C46—H46	120.1
C7—C11—C12	118.7 (3)	C45—C46—H46	120.1
N1—C12—C4	121.8 (3)	C46—C47—C48	117.7 (6)
N1—C12—C11	118.8 (3)	C46—C47—H47	121.1
C4—C12—C11	119.4 (3)	C48—C47—H47	121.1
O1—C13—O2	125.3 (3)	N6—C48—C47	124.7 (6)
O1—C13—C14	117.5 (3)	N6—C48—H48	117.6
O2—C13—C14	117.2 (3)	C47—C48—H48	117.6
C19—C14—C15	118.5 (3)	N6—C49—C45	121.1 (5)
C19—C14—C13	120.4 (3)	N6—C49—C50	118.3 (4)
C15—C14—C13	121.0 (3)	C45—C49—C50	120.6 (4)
C14—C15—C16	120.8 (3)	N5—C50—C42	123.0 (5)
C14—C15—H15	119.6	N5—C50—C49	118.7 (4)
C16—C15—H15	119.6	C42—C50—C49	118.2 (4)
C17—C16—C15	119.8 (3)		
			
O2^i^—Pr1—O1—C13	−87.7 (4)	C3—C4—C12—N1	1.9 (5)
O5^ii^—Pr1—O1—C13	64.3 (4)	C5—C4—C12—N1	−178.3 (3)
O4—Pr1—O1—C13	−3.9 (5)	C3—C4—C12—C11	−177.0 (3)
O7—Pr1—O1—C13	−162.2 (4)	C5—C4—C12—C11	2.8 (5)
O8^ii^—Pr1—O1—C13	−6.5 (4)	N2—C11—C12—N1	−2.4 (4)
O8—Pr1—O1—C13	−129.9 (4)	C7—C11—C12—N1	178.8 (3)
N2—Pr1—O1—C13	132.3 (4)	N2—C11—C12—C4	176.5 (3)
N1—Pr1—O1—C13	133.2 (4)	C7—C11—C12—C4	−2.2 (5)
O2^i^—Pr1—O4—C20	−42.3 (3)	Pr1—O1—C13—O2	−22.6 (6)
O5^ii^—Pr1—O4—C20	170.6 (3)	Pr1—O1—C13—C14	157.1 (3)
O1—Pr1—O4—C20	−118.4 (3)	Pr1^ii^—O2—C13—O1	6.1 (7)
O7—Pr1—O4—C20	36.5 (4)	Pr1^ii^—O2—C13—C14	−173.6 (4)
O8^ii^—Pr1—O4—C20	−115.7 (3)	O1—C13—C14—C19	170.8 (3)
O8—Pr1—O4—C20	22.2 (3)	O2—C13—C14—C19	−9.4 (5)
N2—Pr1—O4—C20	93.2 (3)	O1—C13—C14—C15	−8.8 (5)
N1—Pr1—O4—C20	119.8 (3)	O2—C13—C14—C15	171.0 (3)
O2^i^—Pr1—O7—N7	78.52 (19)	C19—C14—C15—C16	0.9 (5)
O5^ii^—Pr1—O7—N7	−117.83 (18)	C13—C14—C15—C16	−179.5 (3)
O1—Pr1—O7—N7	151.84 (19)	C14—C15—C16—C17	−1.3 (6)
O4—Pr1—O7—N7	−14.1 (2)	C15—C16—C17—O3	−179.9 (4)
O8^ii^—Pr1—O7—N7	94.6 (2)	C15—C16—C17—C18	0.3 (7)
O8—Pr1—O7—N7	4.48 (16)	O3—C17—C18—C19	−178.7 (4)
N2—Pr1—O7—N7	−73.06 (19)	C16—C17—C18—C19	1.1 (7)
N1—Pr1—O7—N7	−138.2 (2)	C17—C18—C19—C14	−1.6 (7)
O2^i^—Pr1—O8—N7	−94.08 (18)	C15—C14—C19—C18	0.5 (6)
O5^ii^—Pr1—O8—N7	108.21 (19)	C13—C14—C19—C18	−179.1 (4)
O1—Pr1—O8—N7	−50.1 (2)	Pr1^i^—O5—C20—O4	−57.3 (5)
O4—Pr1—O8—N7	158.81 (18)	Pr1^i^—O5—C20—C21	121.8 (3)
O7—Pr1—O8—N7	−4.39 (16)	Pr1—O4—C20—O5	20.8 (5)
O8^ii^—Pr1—O8—N7	−148.99 (13)	Pr1—O4—C20—C21	−158.3 (2)
N2—Pr1—O8—N7	78.51 (17)	O5—C20—C21—C26	5.2 (4)
N1—Pr1—O8—N7	31.05 (18)	O4—C20—C21—C26	−175.7 (3)
O2^i^—Pr1—O8—Pr1^i^	54.12 (11)	O5—C20—C21—C22	−171.6 (3)
O5^ii^—Pr1—O8—Pr1^i^	−103.59 (13)	O4—C20—C21—C22	7.5 (4)
O1—Pr1—O8—Pr1^i^	98.11 (13)	C26—C21—C22—C23	0.6 (6)
O4—Pr1—O8—Pr1^i^	−52.99 (11)	C20—C21—C22—C23	177.4 (4)
O7—Pr1—O8—Pr1^i^	143.81 (16)	C21—C22—C23—C24	0.1 (7)
O8^ii^—Pr1—O8—Pr1^i^	−0.8 (2)	C22—C23—C24—O6	178.8 (4)
N2—Pr1—O8—Pr1^i^	−133.28 (13)	C22—C23—C24—C25	0.1 (6)
N1—Pr1—O8—Pr1^i^	179.25 (10)	O6—C24—C25—C26	−179.6 (4)
O2^i^—Pr1—N1—C1	−49.4 (3)	C23—C24—C25—C26	−1.0 (6)
O5^ii^—Pr1—N1—C1	98.8 (3)	C22—C21—C26—C25	−1.5 (5)
O1—Pr1—N1—C1	3.9 (3)	C20—C21—C26—C25	−178.3 (3)
O4—Pr1—N1—C1	153.2 (2)	C24—C25—C26—C21	1.7 (5)
O7—Pr1—N1—C1	−96.9 (3)	C38—N3—C27—C28	−1.0 (7)
O8^ii^—Pr1—N1—C1	55.1 (3)	N3—C27—C28—C29	−0.6 (9)
O8—Pr1—N1—C1	−125.0 (3)	C27—C28—C29—C30	1.7 (9)
N2—Pr1—N1—C1	−176.9 (3)	C28—C29—C30—C38	−1.2 (7)
O2^i^—Pr1—N1—C12	139.7 (2)	C28—C29—C30—C31	179.2 (5)
O5^ii^—Pr1—N1—C12	−72.0 (2)	C29—C30—C31—C32	−178.0 (5)
O1—Pr1—N1—C12	−166.9 (2)	C38—C30—C31—C32	2.3 (6)
O4—Pr1—N1—C12	−17.6 (3)	C30—C31—C32—C33	0.1 (7)
O7—Pr1—N1—C12	92.3 (2)	C31—C32—C33—C34	176.6 (4)
O8^ii^—Pr1—N1—C12	−115.8 (2)	C31—C32—C33—C37	−2.5 (6)
O8—Pr1—N1—C12	64.2 (2)	C32—C33—C34—C35	−178.6 (5)
N2—Pr1—N1—C12	12.3 (2)	C37—C33—C34—C35	0.5 (7)
O2^i^—Pr1—N2—C10	63.6 (3)	C33—C34—C35—C36	−2.9 (9)
O5^ii^—Pr1—N2—C10	−106.5 (3)	C37—N4—C36—C35	0.3 (9)
O1—Pr1—N2—C10	−179.9 (2)	C34—C35—C36—N4	2.7 (11)
O4—Pr1—N2—C10	−24.2 (3)	C36—N4—C37—C33	−2.9 (6)
O7—Pr1—N2—C10	105.4 (3)	C36—N4—C37—C38	176.4 (4)
O8^ii^—Pr1—N2—C10	−66.2 (3)	C34—C33—C37—N4	2.6 (6)
O8—Pr1—N2—C10	53.6 (3)	C32—C33—C37—N4	−178.3 (4)
N1—Pr1—N2—C10	179.1 (3)	C34—C33—C37—C38	−176.7 (3)
O2^i^—Pr1—N2—C11	−129.3 (2)	C32—C33—C37—C38	2.4 (5)
O5^ii^—Pr1—N2—C11	60.6 (2)	C27—N3—C38—C30	1.5 (6)
O1—Pr1—N2—C11	−12.8 (3)	C27—N3—C38—C37	−177.0 (4)
O4—Pr1—N2—C11	142.9 (2)	C29—C30—C38—N3	−0.5 (6)
O7—Pr1—N2—C11	−87.5 (2)	C31—C30—C38—N3	179.2 (3)
O8^ii^—Pr1—N2—C11	100.9 (2)	C29—C30—C38—C37	178.0 (4)
O8—Pr1—N2—C11	−139.3 (2)	C31—C30—C38—C37	−2.3 (5)
N1—Pr1—N2—C11	−13.8 (2)	N4—C37—C38—N3	−0.7 (5)
Pr1—O7—N7—O9	170.5 (3)	C33—C37—C38—N3	178.6 (3)
Pr1—O7—N7—O8	−7.8 (3)	N4—C37—C38—C30	−179.3 (3)
Pr1^i^—O8—N7—O9	40.3 (4)	C33—C37—C38—C30	0.0 (5)
Pr1—O8—N7—O9	−170.9 (3)	C50—N5—C39—C40	−0.5 (9)
Pr1^i^—O8—N7—O7	−141.3 (2)	N5—C39—C40—C41	1.9 (11)
Pr1—O8—N7—O7	7.5 (3)	C39—C40—C41—C42	−1.2 (10)
C12—N1—C1—C2	−0.6 (5)	C40—C41—C42—C50	−0.7 (8)
Pr1—N1—C1—C2	−171.5 (3)	C40—C41—C42—C43	179.0 (6)
N1—C1—C2—C3	1.3 (6)	C41—C42—C43—C44	−179.7 (5)
C1—C2—C3—C4	−0.4 (6)	C50—C42—C43—C44	−0.1 (7)
C2—C3—C4—C12	−1.1 (6)	C42—C43—C44—C45	−2.3 (8)
C2—C3—C4—C5	179.1 (4)	C43—C44—C45—C46	−176.7 (5)
C3—C4—C5—C6	178.3 (4)	C43—C44—C45—C49	1.8 (7)
C12—C4—C5—C6	−1.5 (6)	C49—C45—C46—C47	−1.8 (6)
C4—C5—C6—C7	−0.4 (6)	C44—C45—C46—C47	176.7 (4)
C5—C6—C7—C8	−178.6 (4)	C45—C46—C47—C48	2.2 (7)
C5—C6—C7—C11	1.0 (6)	C49—N6—C48—C47	−0.7 (7)
C11—C7—C8—C9	−1.9 (6)	C46—C47—C48—N6	−1.0 (7)
C6—C7—C8—C9	177.7 (4)	C48—N6—C49—C45	1.1 (6)
C7—C8—C9—C10	0.3 (6)	C48—N6—C49—C50	−178.4 (4)
C11—N2—C10—C9	−3.0 (5)	C46—C45—C49—N6	0.1 (6)
Pr1—N2—C10—C9	164.5 (3)	C44—C45—C49—N6	−178.5 (4)
C8—C9—C10—N2	2.3 (6)	C46—C45—C49—C50	179.6 (4)
C10—N2—C11—C7	1.2 (5)	C44—C45—C49—C50	1.0 (6)
Pr1—N2—C11—C7	−166.4 (2)	C39—N5—C50—C42	−1.6 (7)
C10—N2—C11—C12	−177.6 (3)	C39—N5—C50—C49	178.1 (4)
Pr1—N2—C11—C12	14.9 (4)	C41—C42—C50—N5	2.2 (6)
C8—C7—C11—N2	1.2 (5)	C43—C42—C50—N5	−177.4 (4)
C6—C7—C11—N2	−178.4 (3)	C41—C42—C50—C49	−177.5 (4)
C8—C7—C11—C12	179.9 (3)	C43—C42—C50—C49	2.8 (6)
C6—C7—C11—C12	0.4 (5)	N6—C49—C50—N5	−3.5 (5)
C1—N1—C12—C4	−1.1 (5)	C45—C49—C50—N5	177.0 (3)
Pr1—N1—C12—C4	170.2 (2)	N6—C49—C50—C42	176.3 (3)
C1—N1—C12—C11	177.9 (3)	C45—C49—C50—C42	−3.3 (5)
Pr1—N1—C12—C11	−10.9 (4)		

Symmetry codes: (i) *x*, −*y*+3/2, *z*−1/2; (ii) *x*, −*y*+3/2, *z*+1/2.

### Hydrogen-bond geometry (Å, º)

**Table:**

*D*—H···*A*	*D*—H	H···*A*	*D*···*A*	*D*—H···*A*
O6—H6···N4^iii^	0.82	2.07	2.851 (4)	158
O3—H3···N6^iv^	0.82	2.04	2.782 (5)	150

Symmetry codes: (iii) *x*+1, −*y*+3/2, *z*−1/2; (iv) −*x*+1, −*y*+1, −*z*+1.
